# ﻿First record of *Bibrax* Fletcher, 1927 (Coleoptera, Staphylinidae, Pselaphinae) from Ecuador, with descriptions of twelve new species

**DOI:** 10.3897/zookeys.1250.156763

**Published:** 2025-08-26

**Authors:** Yarina Tapuy-Avilés, David R. Díaz-Guevara, Michael S. Caterino

**Affiliations:** 1 Pontificia Universidad Católica de Ecuador, Quito, Ecuador Fundación Uru Quito Ecuador; 2 Fundación Uru, Quito, Ecuador Pontificia Universidad Católica de Ecuador Quito Ecuador; 3 Instituto Nacional de Biodiversidad, Quito, Ecuador Instituto Nacional de Biodiversidad Quito Ecuador; 4 Clemson University, South Carolina, USA Clemson University Clemson United States of America

**Keywords:** Leaf litter beetles, microphthalmy, tropical biodiversity

## Abstract

Twelve new species of *Bibrax* Fletcher, 1927 (Coleoptera: Staphylinidae: Pselaphinae: Euplectitae: Metopiasini) from Ecuador are described: *B. chullachaqui***sp. nov.**, *B. chocoensis***sp. nov.**, *B. onorei***sp. nov.**, *B. aratrifer***sp. nov.**, *B. longiventer***sp. nov.**, *B. cerroblanco***sp. nov.**, *B. canelazo***sp. nov.**, *B. pectinifer***sp. nov.**, *B. arachnoides***sp. nov.**, *B. amasanga***sp. nov.**, *B. yasuni***sp. nov.**, and *B. grandis***sp. nov.** A key for all species of *Bibrax* is provided. These are the first records of the genus for the country, and we report species from most major environments in the country, from seasonal coastal forests to cloud forests and the Amazonian Basin. The new species expand the scope of morphological variability in the genus, with discovery of numerous microphthalmous and wingless species, and a range of previously unreported secondary sexual characters. Most of the species are only known from single localities, suggesting a large fauna remaining to be discovered from a large portion of South America.

## Abstract (es)

Se describen doce nuevas especies de *Bibrax* Fletcher (Coleoptera: Staphylinidae: Pselaphinae: Euplectitae: Metopiasini) del Ecuador: *B. chullachaqui***sp. nov.**, *B. chocoensis***sp. nov.**, *B. onorei***sp. nov.**, *B. aratrifer***sp. nov.**, *B. longiventer***sp. nov.**, *B. cerroblanco***sp. nov.**, *B. canelazo***sp. nov.**, *B. pectinifer***sp. nov.**, *B. arachnoides***sp. nov.**, *B. amasanga***sp. nov.**, *B. yasuni***sp. nov.**, and *B. grandis***sp. nov.** Se proporciona una clave para todas las especies de *Bibrax*. Estos son los primeros registros del género para el país y reportamos especies de la mayoría de los principales ecosistemas o hábitat del país, desde bosques costeros estacionales hasta bosques nubosos y la cuenca amazónica. Las nuevas especies amplían el alcance de la variabilidad morfológica del género, con numerosas especies microftálmicas y no voladoras, y una gama de caracteres sexuales secundarios no reportados previamente. La mayoría de las especies son endémicas de localidades individuales, lo que sugiere que aún queda una gran fauna por descubrir en gran parte de Sudamérica.

## ﻿Introduction

Pselaphinae are one of the most species-rich and taxonomically neglected groups of rove beetles in the neotropics. Among Neotropical Pselaphinae, however, a few groups have received more attention than others, including the euplectite tribe Metopiasini. The Metopiasini tend to be larger-bodied than most pselaphines, and have striking morphological characters, such as elongated legs and antennae. These characters have helped make them easier to recognize as a group, and more straightforward to work with morphologically.

Starting with a series of publications by André Comellini, most of the genera of this tribe have been subjected to modern revisions, including *Metopioxys* Reitter, 1885 (Comellini 1983), *Metopias* Gory, 1832 (Comellini 1993), *Metopiosoma* Raffray, 1908 (Comellini 1994), *Macrommatias* Gaudin & Coache, 2022 (new name for *Chandleria* Comellini, 1998; Gaudin and Coache 2022), and *Metopiasoides* Comellini, 2000 (Comellini 2000). More recent work has seen new species descriptions in the genera *Metopiasoides* (Asenjo 2016), *Metopiellus* Raffray, 1908 (Asenjo et al. 2017, 2023; Fiorentino et al. 2022; Mario Chaul and Lopes-Andrade 2024), *Metopioxys* (Asenjo et al. 2019), and finally, the subject of this paper, *Bibrax* (Asenjo et al. 2024).

*Bibrax*, originally described by Fletcher (1927) from Panama, currently contains two species, *B. bradleyi* Fletcher, 1927 described with the genus, and *B. popeye*Asenjo et al., 2024 from Colombia. Despite its obvious similarity with other Metopiasini, it has spent much of its taxonomic history in the Goniacerini (see, e.g., Comellini 1979), where it was first placed by Fletcher. Among the characters considered to exclude it from Metopiasini by Fletcher were the lack of divided last male ventrite, possession of only a single tarsal claw, and a completely exposed first abdominal ventrite. Park (1942) explicitly agreed with this arrangement. In Newton and Chandler’s (1989) catalog of the genera of ‘Pselaphidae’, *Bibrax* was united with other ‘Metopiini’ (later renamed to the current Metopiasini; Newton and Thayer 1992), although the tribe was placed in ‘Batrisinae’ (now supertribe Batrisitae). Then the whole tribe was joined with Rhinoscepsina in the Euplectitae by Chandler (2002), where Metopiasini now sits. The character conflicts inherent in these various placements, however, have received little explicit attention.

In this paper we describe several new species of *Bibrax* from Ecuador, providing evidence that the two previously known species of the genus only scratched the surface of its diversity. The new species range from lowland sites on both coastal and Amazonian slopes to 2500 m in the Andean cloud forests. They also encompass considerable morphological diversity, which will be helpful in finding this group’s rightful place in pselaphine classification.

## ﻿Materials and methods

All newly collected specimens were obtained by sifting forest floor leaf litter through an 8-mm mesh. Sifted litter was processed using Berlese/Tullgren and Winkler extractors, with specimens collected and stored in 95% EtOH. One specimen of each morphospecies was prepared for DNA barcoding by poking a small hole at the base of the abdomen with a fine pin. The specimen was then dried and extracted using a HotShot protocol (e.g., Guzmán-Larralde et al. 2017). We attempted to amplify the barcoding region of the mitochondrial Cytochrome Oxidase I using primers mcLCO1460 (KGTCAACAAAYCAYAARGAYATTGG) and BR2 (TCDGGRTGNCCRAARAAYCA; Elbrecht and Leese 2017). However, no amplifications were successful, so we do not address this further. Existing DNA extractions remain in the collection of Instituto Nacional de Biodiversidad (INABIO).

Voucher specimens were mounted, labelled as such (with DNA extraction numbers) and included in type series. All specimens are deposited in the following collections: **QCAZ** – Museo de Zoologia, Pontificia Universidad Católica del Ecuador, Quito; **MECN** – Instituto Nacional de Biodiversidad, Quito.

Species recognition was based on unique combinations of external and male genitalic characters. Most *Bibrax* species exhibit pronounced male secondary sexual characters, although these are found on an unusual variety of body parts, from the antennae to the legs to the abdominal ventrites. These provided initial hypotheses of species distinctness, with aedeagal characters used to test these hypotheses.

Morphological terminology largely follows that used by Asenjo et al. (2024) in their recent description of *B. popeye*, which largely follows Chandler (2001). We refer to the ‘diaphragmatic sclerite’ where a distinct sclerite is visible within the dorsal diaphragm of the aedeagal basal bulb. This more or less circular region may simply be membranous within. Formal descriptions below include only body length (an average of available measurements), while all body measurements (following Asenjo et al. 2024) are provided in Table 1.

**Table 1.** Measurements of all species of *Bibrax*, in millimeters (mm).

Species	AL	BL	BW	EL	EW	HL	HW	NW	PL	PW
*B. arachnoides*	1.32	2.38	0.72	0.65	0.72	0.58	0.43	0.21	0.48	0.47
*B. arachnoides*	1.28	2.31	0.68	0.63	0.68	0.55	0.40	0.22	0.48	0.46
*B. arachnoides*	1.22	2.26	0.66	0.57	0.66	0.56	0.39	0.21	0.48	0.44
*B. arachnoides* (♀)	1.20	2.17	0.64	0.55	0.64	0.52	0.38	0.22	0.45	0.44
*B. amasanga*	1.70	2.89	0.80	0.80	0.80	0.63	0.46	0.25	0.56	0.57
*B. yasuni*	1.42	2.44	0.71	0.68	0.71	0.51	0.39	0.24	0.51	0.50
*B. chullachaqui*	1.70	2.83	0.92	0.94	0.92	0.56	0.51	0.24	0.57	0.58
*B. pectinifer*	1.13	2.02	0.57	0.52	0.57	0.48	0.34	0.19	0.41	0.40
*B. pectinifer*	1.15	2.06	0.56	0.50	0.56	0.53	0.36	0.21	0.38	0.37
*B. pectinifer* (♀)	1.14	2.08	0.56	0.50	0.56	0.54	0.32	0.18	0.40	0.39
*B. longiventer*	1.71	2.65	0.74	0.58	0.74	0.58	0.47	0.21	0.36	0.53
*B. longiventer*	1.56	2.49	0.72	0.63	0.72	0.52	0.46	0.21	0.41	0.53
*B. longiventer* (♀)	1.45	2.28	0.73	0.58	0.73	0.43	0.46	0.22	0.40	0.53
*B. chocoensis*	1.46	2.52	0.73	0.75	0.73	0.57	0.43	0.22	0.49	0.46
*B. grandis* (♀)	1.79	2.58	0.85	0.56	0.85	0.36	0.47	0.23	0.43	0.58
*B. canelazo*	0.83	1.45	0.47	0.35	0.47	0.36	0.31	0.15	0.26	0.35
*B. canelazo* (♀)	1.13	1.75	0.45	0.33	0.45	0.35	0.30	0.15	0.27	0.34
*B. cerroblanco*	1.49	2.54	0.77	0.66	0.77	0.55	0.40	0.22	0.50	0.47
*B. cerroblanco*	1.48	2.54	0.81	0.62	0.81	0.56	0.42	0.22	0.50	0.48
*B. cerroblanco* (♀)	1.45	2.52	0.79	0.66	0.79	0.54	0.42	0.21	0.53	0.50
*B. onorei*	0.80	1.38	0.48	0.30	0.48	0.31	0.32	0.16	0.27	0.35
*B. onorei*	0.78	1.41	0.46	0.33	0.46	0.34	0.31	0.16	0.29	0.36
*B. onorei* (♀)	0.83	1.46	0.47	0.33	0.47	0.36	0.32	0.16	0.27	0.37
*B. aratrifer*	0.94	1.66	0.50	0.36	0.50	0.38	0.29	0.15	0.34	0.35
*B. aratrifer*	0.96	1.69	0.54	0.36	0.54	0.39	0.34	0.17	0.34	0.37
*B. aratrifer* (♀)	0.97	1.70	0.55	0.38	0.55	0.39	0.32	0.16	0.34	0.38

Abbreviations used in the text are as follows:

**AL** abdomen length

**BL** body length (from the anterior margin of the prolongation of the head to the posterior margin of tergite VIII)

**BW** body width (maximum width of elytra in dorsal view)

**EL** elytral length (maximum in dorsal view)

**EW** elytral width (maximum in dorsal view)

**HL** head length (from the anterior margin of the prolongation of the head to the posterior margin of the head disc in frontal view)

**HW** head width (maximum, including eyes, in frontal view)

**NW** neck width (minimum in dorsal view)

**PL** pronotum length (maximum in dorsal view)

**PW** pronotum width (maximum in dorsal view)

## ﻿Taxonomy

### ﻿Checklist of all species of Bibrax

Male eyes large (>10 ommatidia present):

1. *B. popeye* Asenjo et al., 2024
2. *B. chullachaqui* sp. nov.
3. *B. chocoensis* sp. nov.

Male eyes small (<<10 ommatidia, usually <4):

Antennae (pedicel) not dimorphic:

1. *B. bradleyi* Fletcher, 1927 (type species)
2. *B. onorei* sp. nov.
3. *B. aratrifer* sp. nov.
4. *B. longiventer* sp. nov.
5. *B. cerroblanco* sp. nov.
6. *B. canelazo* sp. nov.
7. *B. pectinifer* sp. nov.

Antennae (pedicel) dimorphic:

1. *B. arachnoides* sp. nov.
2. *B. amasanga* sp. nov.
3. *B. yasuni* sp. nov.

Male eyes unknown (female only):

1. *B. grandis* sp. nov.

### ﻿Key to males (males either have large eyes and fully developed flight wings, or they exhibit secondary modifications of the antennal pedicel, abdominal ventrites, metatrochanters, or tibial apices)

**Table:** 

1	Pronotum with well-developed lateral lobes, strongly constricted posteriorly (Figs 3A, E, 4A, 6A, E, 8F)	**9**
–	Pronotum without well-developed lateral lobes, more gradually widened from base (Figs 1A, D, 4E, 7A, E, 8A)	**2**
2	Eyes well-developed, comprising >4 ommatidia	**3**
–	Eyes strongly reduced, with ≤4 ommatidia	**5**
3	Antennal pedicel (antennomere II) swollen, much wider than III (Fig. 1A–C); body setae uniformly short, appressed	***B. chullachaqui***
–	Antennal pedicel unmodified, little wider than antennomere III; body setae tending to be longer	**4**
4	Metatibia apically bifid, with strong inner marginal tooth; pronotum little longer than wide; postfoveal sulci of elytra longer than half of elytral length (Asenjo et al. 2024: figs 1, 2)	***B. popeye***
–	Metatibia undivided; pronotum distinctly longer than wide; postfoveal sulci of elytra shorter, not extending beyond elytral midpoint (Fig. 1D)	***B. chocoensis***
5	Antennal pedicel (antennomere II) widened along inner margin, much wider at apex than antennomere III (Figs 7A, E, 8A); metatrochanter dentate (Figs 7D, H, 8E)	**6**
–	Antennal pedicel unmodified, little wider than antennomere III; metatrochanter unmodified	**8**
6	Last abdominal ventrite short, with large median tooth on apical margin and smaller median tooth on basal margin, penultimate ventrite unmodified; metatrochanter quadrate, with distal corner produced as a small perpendicular denticle (Fig. 7D); antennal pedicel more simply expanded at inner apical corner, apex truncate (Fig. 7C)	***B. arachnoides***
–	Last abdominal ventrite slightly longer, with median tooth on apical margin, but basal portion simply depressed, penultimate ventrite also depressed; metatrochanter either with small median tooth, or prolonged beneath femur and apically acute; antennal pedicel more swollen along outer edge, inner apical corner expanded into short flange beyond articulation with antennomere III	**7**
7	Penultimate abdominal ventrite relatively long, distinctly depressed (Fig. 7G); metatrochanter oval, with median posterior tooth (Fig. 7H); antennal pedicel distinctly swollen along outer margin, inner apex not modified (Fig. 7G)	***B. amasanga***
–	Penultimate ventrite shorter, barely depressed at middle; metatrochanter prolonged beneath femur and apically acute (Fig. 8E); antennal pedicel straight along outer margin, with flattened and prolonged spatulate process at inner apex (Fig. 8C)	***B. yasuni***
8	Elytral humeri rounded (winged); male metatibia with inner marginal tooth	***B. bradleyi***
–	Elytra evenly widened from base to apex (wingless); male metatibia lacking tooth along inner margin	***B. cerroblanco***
9	Antennal base not strongly prolonged; frons with lyriform carinae (Fig. 8F)	***B. grandis***
–	Antennal base elongate; frons lacking lyriform carinae	**10**
10	Last abdominal ventrite longer than all other ventrites together, depressed (Fig. 4C)	***B. longiventer***
–	Last abdominal ventrite similar in length to preceding ventrites, no longer than preceding two combined	**11**
11	Second visible abdominal ventrite with curved, scoop-like ventral process, setose along its margin (Fig. 3H)	***B. aratrifer***
–	Second visible abdominal ventrite without curved, scoop-like process, if modified, simply flattened medially	**12**
12	Penultimate abdominal ventrite elevated on each side, with combs of erect setae along margins (Fig. 6H); combs of setae also present on ultimate ventrite	***B. pectinifer***
–	Last two abdominal ventrites lacking lateral combs of setae	**13**
13	Abdominal ventrites 2–4 widely and distinctly depressed medially (Fig. 3D)	***B. onorei***
–	Abdominal ventrites 2–3 unmodified, penultimate ventrite depressed along apical half, and ultimate with small median tooth (Fig. 6D)	***B. canelazo***

### ﻿Species descriptions

#### ﻿ Bibrax

Taxon classificationAnimaliaColeopteraStaphylinidae

Fletcher, 1927

B4E0DBE1-8327-5AF6-9739-B85FDCAC0924

##### Type species.

*Bibrax bradleyi* Fletcher, 1927, by original designation. Type locality. Barro Colorado Island, Panama.

##### Diagnosis.

Among Metopiasini, *Bibrax* has been diagnosed (e.g., by Asenjo et al. 2024; Mario Chaul and Lopes-Andrade 2024; Chandler, unpublished) by having a scape as long as the funicular antennomeres combined (unlike *Rhinoscepsis*), the abdomen laterally margined, with distinct paratergites (unlike *Metopioxys* and *Metopiosoma*), the second antennomere or pedicel (at least up until now) at least 2× as long as the third, the lack of acute pronotal and vertexal tubercles (seen in *Metopiellus*), having distinct lateral and/or median longitudinal pronotal sulci, having abdominal sternite III (first visible) longer than the metacoxae, and having the anterolateral corners of the gula acutely projecting (the size of which are, in light of new species, quite varied).

Secondary sexual characters, when present, frequently involve modifications of the male abdominal sternites, the male tibial apices, male antennal pedicel, and male trochanters. In some species the sexes are dimorphic in eye size, although in several species described below reduced eyes are present in both sexes. The male genitalia, while highly varied in particulars, are consistent in having a large basal bulb that is articulated with an elongate distal tegmen. Parameres are absent. Asenjo et al (2024) demonstrated the basal articulation of the basal bulb with an “apodemal plate” on the inner surface of sternite VIII, and all of the species have a variously developed process (sometimes paired) on the basal bulb for this purpose. The tegmen can be simple or quite elaborate, sometimes subdivided, and is highly diagnostic of each species.

#### ﻿ Bibrax chullachaqui sp. nov.

Taxon classificationAnimaliaColeopteraStaphylinidae

2E7D8CFD-B4D1-5B2C-AB57-E0731658DCC3

https://zoobank.org/284E9DF9-9727-489A-9265-9758F329B285

Figs 1A–C , 2A, B

##### Type material.

***Holotype*** • ♂ (MECN-EN 23779): “Ecuador. Napo, Archidona. Pacto Sumaco, -0.658219, -77.59197. Malaise, 22-ene-2024. A Pazmiño | M Barreno” / “Caterino DNA voucher, Ext. MSC-13221, Morphosp. PS.A.003” / “MECN-EN 23779”.

**Figure 1.**
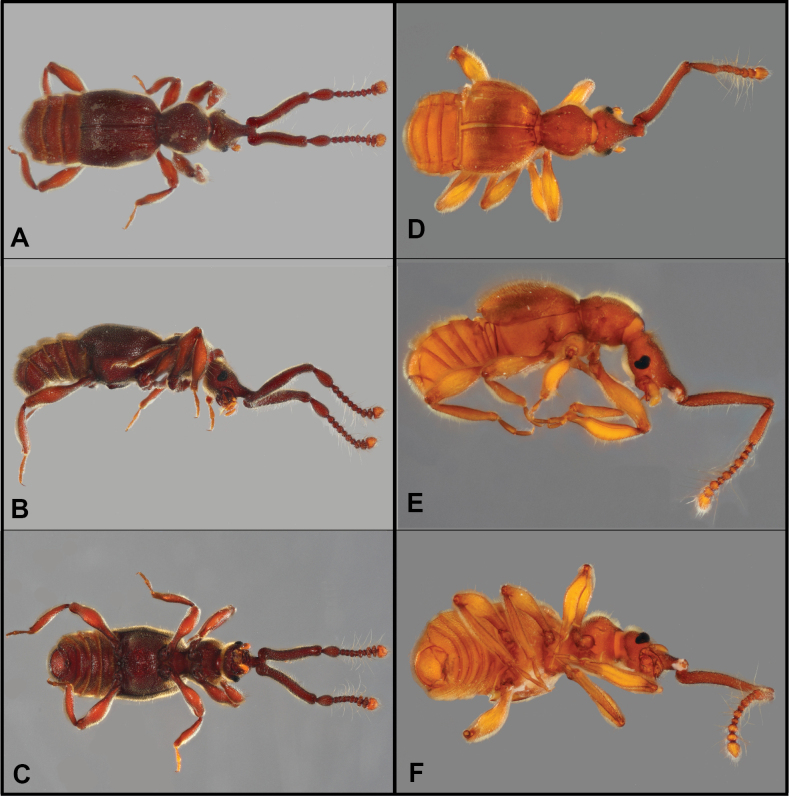
Habitus of *Bibrax* species (A, D. Dorsal view; B, E. Lateral view; C, F. Ventral view). A–C. *B. chullachaqui* sp. nov.; D–F. *B. chocoensis* sp. nov.

##### Diagnosis.

**Figure 2.**
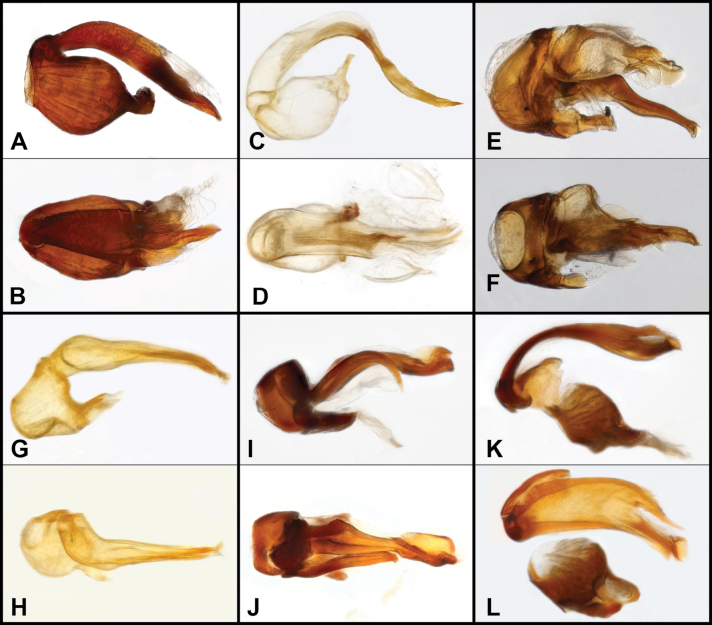
Aedeagus of *Bibrax* species, lateral view (A, C, E, G, I, K) and dorsal view (B, D, F, H, J, L). A, B. *B. chullachaqui* sp. nov.; C, D. *B. chocoensis* sp. nov.; E, F. *B. onorei* sp. nov.; G, H. *B. aratrifer* sp. nov.; I, J. *B. longiventer* sp. nov.; K, L. *B. cerroblanco* sp. nov.

BL = 1.89 mm (*n* = 1); body densely setose and brownish-orange; eyes prominent, borne on small prominence, comprising ~26 ommatidia; gular teeth well-developed, curved slightly dorsad; head rounded posteriorly, prolonged in front of eyes to prominent antennal base, vertexal foveae slightly impressed; frons finely punctate above; antennal scape thick and sinuate, slightly narrowed before apex (in lateral view), antennomere II (pedicel) swollen, approximately as long as III–V together, III–V similar in size, short, VI and VIII shorter, VII wider than long, IX–XI forming a club, IX and X transverse, X slightly wider than IX, X, and XI closely associated; pronotum rounded, widest anterad, with lateral lobes slightly produced; lateral longitudinal pronotal impressions very shallowly impressed; elytra longer than wide, slightly widened beyond midpoint, each with sutural and one lateral basal foveae, lateral with shallow longitudinal impression extending posteriorly ~2/3 elytron length; subhumeral fovea absent; flight wings present protibia swollen; metatrochanter not modified, metaventrite between second and third pair of legs with oblique carinae posterad a shallow median depression; last abdominal ventrite bearing small median apical marginal flange; third ventrite slightly flattened, posterior margin straight at middle. Aedeagus (Fig. 2A, B) with basal bulb large, elongate oval, with blunt, apically knobbed pair of basal apodemes; diaphragm rather small, round, located just basad tegmen articulation; tegmen widened from base as it curves dorsad and distad, apex divided, with an elongate, sinuate subacute process extending beneath shorter, broad dorsal brush.

##### Distribution.

This species is known only from the lower (~1500 m), southern slopes of the Volcán Sumaco, in the western Amazon, Napo Province, Ecuador.

##### Remarks.

This species is most similar to *B. bradleyi* and *B. popeye* in having well-developed eyes and flight wings (at least in the male sex), while having the pronotum rather simply expanded toward the front, with only weak longitudinal sulci. It is distinctive in its dense covering of short, stiff setae, while the short swollen second antennomere and compact antennal club are also distinctive. While lacking any secondary sexual characters on the legs, the depressed male metaventrite is unusual.

##### Etymology.

The species is named after Chullachaki, a mythical ‘one-footed’ spirit of the Amazonian jungle. He is purportedly able to change into any type of rainforest animal, guarding the forest against unwise uses.

#### ﻿ Bibrax chocoensis sp. nov.

Taxon classificationAnimaliaColeopteraStaphylinidae

2E8A8F1D-989E-58C8-9F7E-907D80309570

https://zoobank.org/63B6847B-212F-4FA7-A86C-3AF732F58C9B

Figs 1D–F , 2C, D

##### Type material.

***Holotype*** • ♂ (MECN-EN 40872): “Ecuador: Esmeraldas, 0.56759, -79.06351, Quinindé. Reserva Biológica Canandé. 400 m. 14-oct-2022. Winkler., A Pazmiño | D Díaz.” / “Caterino DNA voucher, Ext. MSC-13015, Morphosp. Can.A.001” / “MECN-EN 40872”.

##### Diagnosis.

BL = 1.79 mm (*n* = 1). Body densely setose, densely and finely punctate beneath; head rounded at base, vertexal foveae shallowly impressed; frons elevated, elongate to common antennal base, with shallow median sulcus on surface; eyes (of male) well developed, convex, with >20 fine facets; gular teeth well-developed; antennae with scape very long, slightly sinuate, antennomere II (pedicel) as long as III-VI combined, III–VIII all bead-like, but V and VII larger, IX–XI forming loose club; pronotum elongate, widest near front, with lateral and median subbasal foveae, as well as row of small secondary foveae along basal margin; disk with lateral and median longitudinal impressions; elytra moderately elongate (winged) with sides rounded, each elytron with sutural and lateral foveae, lateral fovea with strong impression running posterad ~1/2 elytron length; subhumeral fovea absent; protibia swollen, with modified inner marginal spurs; last abdominal ventrite slightly flattened, densely setose on each side. Aedeagus (Fig. 2C, D) with large basal bulb bearing prominent basal flange where it articulates with sternite VI; tegmen articulated with basal bulb, tubular at base, strongly arched dorsad, then curved distad and flattening and narrowing to subacute apex.

##### Distribution.

This species is known only from the coastal forests of Esmeraldas, Ecuador.

##### Remarks.

This species more closely resembles the described species, *B. bradleyi* in particular, in having large male eyes, apparently well-developed flight wings (at least in the male), and pronotum without lobed sides. Its pronotum is more elongate than either *B. bradleyi* or *B. popeye*, and it exhibits distinct secondary sexual characters of the protibial spurs, while lacking metatibial modifications.

##### Etymology.

The name of this species refers to the Chocó biodiversity hotspot, a biome largely limited to northwestern Ecuador and neighboring Colombia, where this species is found.

#### ﻿ Bibrax onorei sp. nov.

Taxon classificationAnimaliaColeopteraStaphylinidae

512A3026-9CAC-5FE9-8BAB-9DE0F3D2D47A

https://zoobank.org/9682E54B-73FE-4DC6-BD7D-7C035C960936

Figs 2E, F , 3A–D

##### Type material.

***Holotype*** • ♂ (MECN-EN 40873): “Ecuador: Cotopaxi, -0.4176, -79.0040, Bosque Integral Otonga, 13.VIII.2024, 2063 m, M. Caterino & A. Pazmiño, Sifted leaf litter” / “Caterino DNA voucher, Ext. MSC-12844, Morphosp. Ot.A.009” / “MECN-EN 40873”. ***Paratypes*** (3♂, 5♀, same general locality as type) • 2: same data as type • 3: -0.4167, -79.0043, 2097 m • 1: -0.4192, -79.0031, 1966 m • 1: -0.4172, -79.0040, 2073 m • 1: -0.4169, -79.0042, 2086 m (MECN-EN 38587-38593, 40897).

##### Diagnosis.

**Figure 3.**
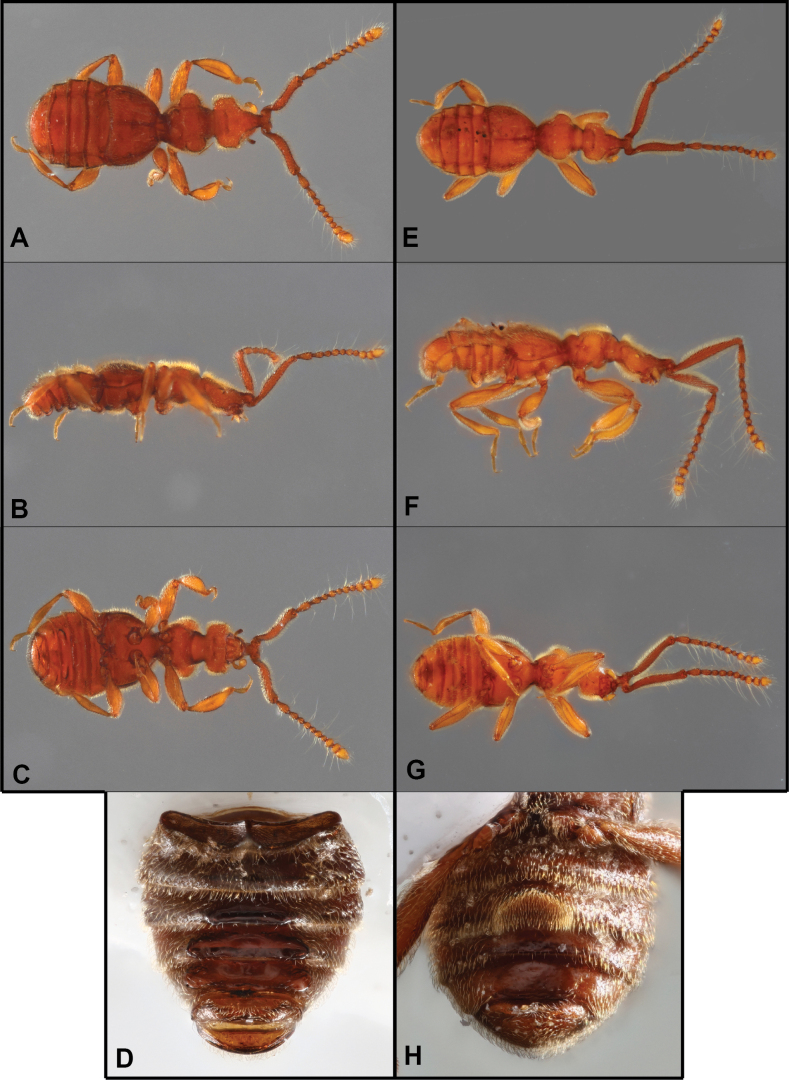
Habitus of *Bibrax* species (A, E. Dorsal view; B, F. Lateral view; C, G. Ventral view) D, H. Male abdominal ventrites). A–D. *B. onorei* sp. nov.; E–H. *B. aratrifer* sp. nov.

BL = 1.10 mm (*n* = 3). Eyes of both sexes with single facet, head subquadrate posteriorly, with transverse basal ridge on vertex, prolonged in front of eyes; antennal base prolonged and narrowed anterad, antennal insertions swollen; antennal scape sinuate, narrowed to base and before apex, antennomere II (pedicel) slightly swollen, ~1.5× as long as III (Fig. 3A–C), III conical, IV–VIII bead-like, alternating slightly in size (IV, VI, VIII smaller), IX–XI forming club, X and XI more closely associated than IX with X; gular processes evident but not large; pronotum with anterior lobes rounded (Fig. 3A), strongly constricted posteriorly; lateral longitudinal sulci conspicuous; median pronotal sulcus weak; elytra very short, together emarginate along posterior margin; dorsal elytral sulci short and weakly developed; abdominal paratergites with posterior corners angulate; male with visible abdominal ventrites 2–4 depressed medially (Fig. 3D); male lacking modification of mesotibial apex. Aedeagus (Fig. 2E, F) with short, broad basal bulb, with oval dorsobasal diaphragm, diaphragmatic sclerite indistinct; short digitiform process extending from left, middle edge of basal bulb; slightly longer, narrow apically quadrate process extending from medioventral margin; longest process extending out of middle of basal bulb (articulated), a well sclerotized, basally bent, apically narrowed and slightly sinuate blade, terminating in blunt and weakly tufted apex; dorsally an articulated, weakly sclerotized process extends over basal two-thirds of median blade.

##### Distribution.

This species is known only from the higher elevations of the Bosque Integral Otonga, in northern Cotopaxi province, Ecuador.

##### Remarks.

This species resembles several other new species described here, all of which differ considerably from any previously described ones. The species in this group (*B. bradleyi*, *B. aratrifer*, *B. longiventer*, *B. cerroblanco*, *B. canelazo*, *B. pectinifer*, *B. arachnoides*, *B. amasanga*, and *B. yasuni*) share strongly reduced eyes and lack of wings in both sexes, a small, somewhat flattened body, strongly impressed lateral longitudinal pronotal sulci that set off distinct lateral pronotal lobes, and exhibit modified abdominal ventrites in the males (Fig. 3D). It is principally in the latter characters (as well as marked differences in male genitalia (Fig. 2E, F) that distinguish the species.

##### Etymology.

This species name honors Dr. Giovanni Onore, a pioneer of entomology in Ecuador, who facilitated the fieldwork and worked to protect the site that led to the discovery of this species.

#### ﻿ Bibrax aratrifer sp. nov.

Taxon classificationAnimaliaColeopteraStaphylinidae

F6DB8F00-AF39-5D00-BA52-115553932086

https://zoobank.org/3BF8B08C-D0C5-4A9C-8010-F934CD7033DC

Figs 2G, H , 3E–H

##### Type material.

***Holotype*** • ♂ (MECN-EN 38735): “Ecuador: Napo, -0.5952, -77.8994, Est. Biol. Yanayacu, Piha Tr., 2386 m, 9.IX.2024, sifted litter, M.Caterino” / “MECN-EN 38735”. ***Paratypes*** (4♂, 1♀, same general locality as type) • 1: same data as type • 1: -0.5983, -77.8954, Stream Tr., 2192 m • 1: -0.5986, -77.8949, Stream Tr., 2185m • 1: -0.5958, -77.8927, Aburrian Tr., 2283 m • 1: -0.5947, -77.8945, Piha Tr., 2248m (MECN-EN 38736-38739, 41045).

##### Diagnosis.

BL = 1.32 mm (*n* = 3). Eyes of both sexes with single facet, head subquadrate posteriorly, prolonged in front of eyes, deeply depressed at vertexal foveae, slightly elevated posteriorly; antennal base prolonged and narrowed anterad; antennal scape sinuate, narrowed to base and before apex, flagellomeres II–VI progressively shorter, VII slightly larger, VIII small, IX–XI forming weak, loose club; pronotum with lateral lobes rounded, median and lateral longitudinal pronotal sulci well developed; elytra very short (flightless in both sexes), together emarginate along posterior margin, each with single distinct, median basal fovea, with short longitudinal elytral sulci short extending posterad; male with visible abdominal ventrite 2 elevated at middle, elevation posteriorly concave, densely setose; male ventrites 3 and 4 simply depressed medially; other secondary sexual characters not evident. Aedeagus (Fig. 2G, H) with large basal bulb with round dorsal diaphragm, bulb ventrally bearing strong basal shelf (which articulates internally with sternite 6); tegmen constricted at base, appearing articulated with basal bulb, bent strongly laterad, thence narrowing evenly distad, apex with very small spatulate tip.

##### Distribution.

This species is known only from the cloud forests of the Yanayacu Biological Station, Napo province, Ecuador.

##### Remarks.

Among the smaller, flattened, and flightless species mentioned previously, the unique, elevated, curved transverse ridge on male ventrite 2 easily distinguish this species.

##### Etymology.

The name of this species refers to this abdominal modification, meaning in Latin ‘plow-bearing’.

#### ﻿ Bibrax longiventer sp. nov.

Taxon classificationAnimaliaColeopteraStaphylinidae

F90D17D8-E113-52CB-B860-39883BA851B7

https://zoobank.org/AD54522D-D080-4CA9-A53D-3916C8BA78CD

Figs 2I, J , 4A–D

##### Type material.

***Holotype*** • ♂ (MECN-EN 40723): “Ecuador: Napo, -0.61523, -77.59071, Archidona, Pacto Sumaco, 1805m, 27.IX.2024, A.Pazmiño & D.Díaz, Winkler” / “Caterino DNA voucher, Ext. MSC-13068, Morphosp. VS.007” / “MECN-EN 40723”. ***Paratypes*** (2♂ 2♀) • same data as type (MECN-EN 40639, 40608, 40651, 40655).

##### Diagnosis.

BL = 1.88 mm (*n* = 3). Body rather large, densely setose, reddish-orange; eyes of both sexes strongly reduced, borne on small prominence, comprising two or three indistinct ommatidia; head subquadrate posteriorly, prolonged in front of eyes to prominent antennal base, deeply depressed at vertexal foveae, slightly elevated posteriorly; antennal scape sinuate, narrowed to base and before apex, flagellomeres II–VI progressively shorter, VII slightly larger, VIII small, IX–XI forming weak, loose club; gular teeth not prominent; pronotum with lateral lobes strongly produced, rounded, pronotum strongly constricted posteriorly; median and lateral longitudinal pronotal impressions well developed; elytra rather short, with narrowed humeri (flightless), each with sutural and one lateral basal foveae, lateral fovea with short longitudinal impression extending posterad; abdominal paratergites wide; protibia swollen; male metaventrite with small flat glabrous area between and anterad metacoxae; male abdominal segment 5 markedly elongate, sternite especially, prolonged anterad into emarginate ventrite 4, deeply concave, with small, tubular denticle at basal margin; legs without obvious secondary sexual characters. Aedeagus (Fig. 2I, J) with basal bulb large, basally truncate, dorsally angulate, with long basal apodeme; tegmen comprising two separate processes, a shorter, strongly arched and apically acute rod on left side; right-side process longer, terminating in dorsoventrally flattened, subrectangular plate.

**Figure 4.**
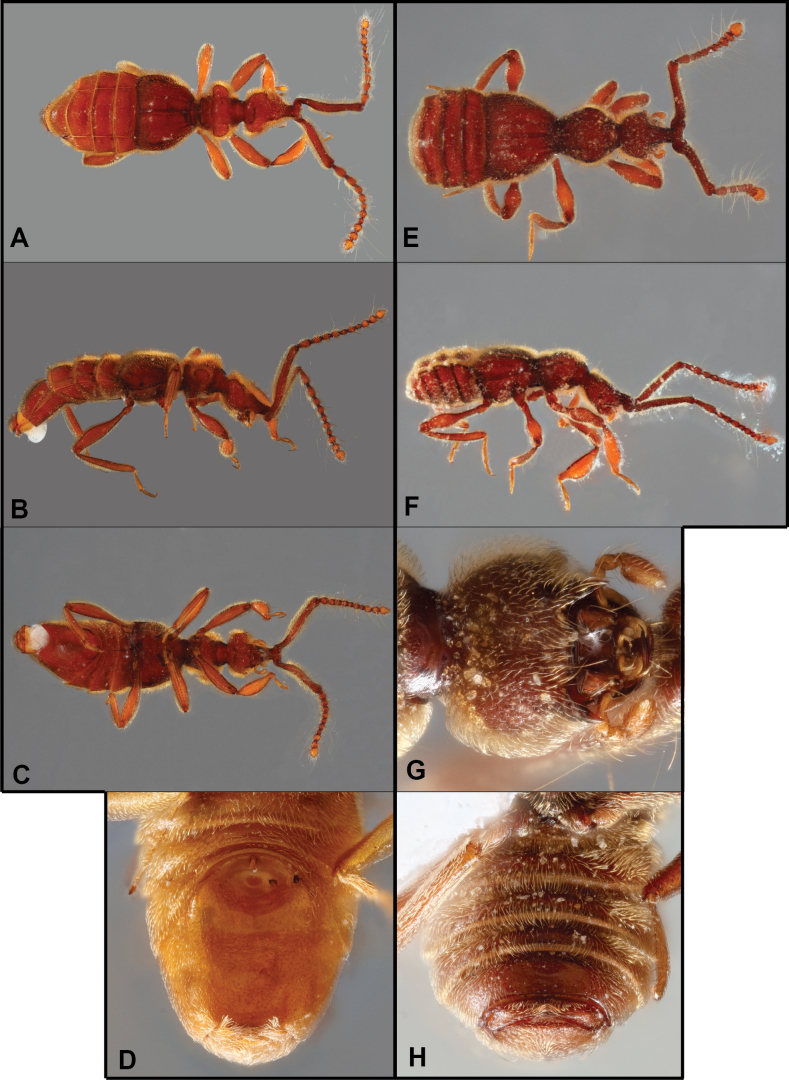
Habitus of *Bibrax* species (A, E. Dorsal view; B, F. Lateral view; C. Ventral view; D, H. Male abdominal ventrites; G. Venter of head). A–D. *B. longiventer* sp. nov.; E–H. *B. cerroblanco* sp. nov.

##### Distribution.

This species is known only from the isolated Volcan Sumaco, in the western Amazonian basin, Ecuador.

##### Remarks.

The remarkably prolonged male sternite 5 in this species is unique among known *Bibrax*, while the strongly laterally lobed (and posteriorly constricted) pronotum may also help distinguish females from those of other species. While among the ‘smaller’, flattened species described herein, this species is also larger than most others.

##### Etymology.

The name of this species refers to the uniquely elongate last abdominal ventrite.

#### ﻿ Bibrax cerroblanco sp. nov.

Taxon classificationAnimaliaColeopteraStaphylinidae

309305BE-D1D5-5649-8EC3-34F52D49FBB4

https://zoobank.org/FA0E16E2-6CD0-4460-8900-6C7C3B0ED0C1

Figs 2K, L , 4E–H

##### Type material.

***Holotype*** • ♂ (MECN-EN 38548): “Ecuador: Guayas, -2.159579, -80.02109, Bosque Protector Cerro Blanco, Q. Cusumbo, 25.IV.2022, Winkler, C. Cujigualpa” / “Caterino DNA voucher, Ext. MSC-13079, Morphosp. CrBl.A.009” / “MECN-EN 38548”. ***Paratypes*** (2♂, 5♀) • same data as type (MECN-EN 38545-38547, 38549-38552).

##### Diagnosis.

BL = 1.89 mm (*n* = 3). Body with dense, matte-like pubescence; head with single large ommatidium on each side (in both sexes); vertexal foveae shallowly impressed; head rounded posteriorly, prolonged anteriorly into narrowly elongate antennal base; gular teeth well-developed; scape very elongate, weakly curved at base, male antennomere II (pedicel), slightly swollen, ~2× as long as III, evenly widened to apex, antennomeres III–V ~1.5× longer than wide, VI–VIII shorter, VII slightly wider than VI or VIII; IX–XI slightly larger, forming loose, weak club; pronotum elongate, ~1.5× longer than wide, widest just anterior of midpoint, evenly narrowed to base and apex; lateral pronotal foveae deeply impressed, setose, joined by weak transverse sulcus that passes through smaller, nude median fovea, continuing laterad to weakly interrupt lateral margin; shallow longitudinal impressions extend anterad and posterad from lateral and median foveae forming weak sulci; elytra narrow at base (wingless), widening to apex, each with sutural and one lateral dorsobasal foveae, shallow sulci extending posterad from foveae; subhumeral fovea absent; wingless in both sexes; protibiae strongly swollen; male ventrite 5 with broad, shallowly concave, smooth median area, male ventrite 6 with minute median, apical tooth. Aedeagus (Fig. 2K, L) with basal bulb large, irregularly subspherical, with prominent basal apodeme; diaphragm located dorsobasally; tegmen dorsoventrally flattened, widened from base as it curves dorsad and distad, apex split ~1/4 from tip, one side with serrate margin narrowing to acute point, other side slightly longer and ending in slightly expanded, blunt apical plate.

##### Distribution.

This species is known only from Ecuador’s coastal mountains just northwest of Guayaquil.

##### Remarks.

This species is among the more convex, less strongly flattened species, having the pronotum gradually narrowed posteriorly, not abruptly constricted behind prominent lateral lobes. Unlike several of these, it does not have male antennomere II expanded, nor does it exhibit modifications of the male metatrochanter. The combination of these, along with a male penultimate metaventrite that is broadly and shallow concave, will distinguish it from any other species currently known. The aedeagus, having a flattened, shallowly bifid tegmen in which the right lobe is marginally denticulate, is highly distinctive.

##### Etymology.

We name this species for the protected area in which it occurs, El Bosque Protector Cerro Blanco. Semi-surrounded by the greater Guayaquil metropolitan area, this oasis of biodiversity has exceptional conservation value.

#### ﻿ Bibrax canelazo sp. nov.

Taxon classificationAnimaliaColeopteraStaphylinidae

86E21074-F99F-5999-8C2E-C0991D6198FF

https://zoobank.org/FA1D89CB-CBAE-42B1-A108-12C2A4FC5C04

Figs 5A, B , 6A–D

##### Type material.

***Holotype*** • ♂ (MECN-EN 40836): “Ecuador: Tungurahua, -1.4361, -78.3105, NaturetrekCandelariaRes, 2241m, 13.XI.2024, M.Caterino, sifted litter” / “Caterino DNA voucher, Ext. MSC-13189, Morphosp. Cnd.005” / “MECN-EN 40836”. ***Paratypes*** (1♂ 1♀, same general locality as type) • 1: -1.4343, -78.3114, 2145m • 1: -1.4361, -78.3105, 2241m (MECN-EN 40332, 40379).

##### Additional material.

• 1♂ (MECN-EN 40794): “Ecuador: Tungurahua, -1.3812, -78.2895, Rio Machay Reserve, 2416m, 12.XI.2024, M.Caterino, sifted litter” / “Caterino DNA voucher, Ext. MSC-13147, Morphosp. Mch.010; (1♂ 8♀, all Rio Machay Reserve) • 3: same data as preceding • 4: -1.3895, -78.2929, 2239m • 1: -1.3801, -78.2889, 2434m • 1: -1.3829, -78.2909, 2382m (MECN-EN 40190, 40206, 40215, 40220, 40229, 40283, 40285, 40292, 40296).

##### Diagnosis.

**Figure 5.**
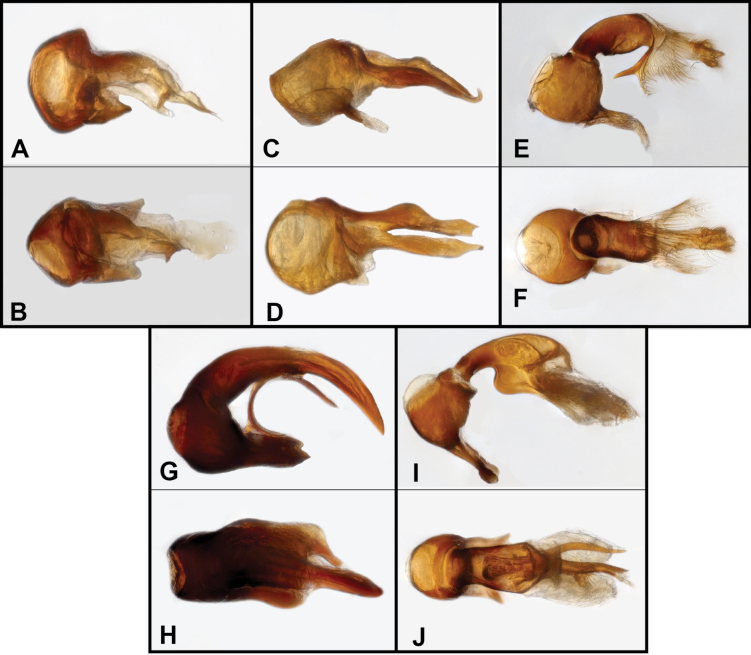
Aedeagus of *Bibrax* species, lateral view (A, C, E, G, I) and dorsal view (B, D, F, H, J). A, B. *B. canelazo* sp. nov.; C, D. *B. pectinifer* sp. nov.; E, F. *B. arachnoides* sp. nov.; G, H. *B. amasanga* sp. nov.; I, J. *B. yasuni* sp. nov.

BL = 1.10 mm (*n* = 2). Body identical in most respects to *B. onorei*, with the following exceptions: antennomeres II-IV slightly more slender, elongate, antennomere III, in particular, nearly as long antennomere II; median longitudinal pronotal sulcus more deeply impressed; male with only last two abdominal ventrites modified, penultimate with weak depression in distal third, weakly margined by transverse carina, last ventrite short, concave, with small slightly transverse apical marginal tooth. Aedeagus (Fig. 5A, B) with basal bulb short, distal surface flat, bearing large oblique diaphragm on left dorsolateral surface; with pair of basal apodemes well separated, obliquely truncate with outer edge longer; tegmen trunk-like, basally cylindrical, extending distad from middle of apical surface of basal bulb, bent ventrad, flattening toward broad paddle-shaped apex; tegmen with short ventral tooth and shorter, weakly sclerotized lobe extending distad below apex of tegmen.

##### Distribution.

This species is known from cloud forests of two localities at similar elevations on either side of the Rio Pastaza in Tungurahua province, Ecuador, both of them reserves managed by the EcoMinga Foundation.

##### Remarks.

There is little to separate this species externally from other small, flattened species described here. It is relatively small, and the male abdominal modifications are comparatively subtle, with only a small distal tooth at the apex of a shallowly depressed last ventrite. There is slight variation in aedeagal shape between the two localities, the ‘apical paddle’ of the type population more elongate, narrower, and apically rounded, while that at Rio Machay is shorter, more quadrate, and pointed at middle. But for the present we prefer to recognize this as intraspecific variation. Discovery of additional populations of either form would allow further assessment of their respective status.

##### Etymology.

The name of this species refers to the popular Ecuadorian beverage, canelazo, made and enjoyed during Fiesta de Quito, an annual festival happening around the time of the discovery of this species (late November). The name also echoes the type locality Candelaria.

#### ﻿ Bibrax pectinifer sp. nov.

Taxon classificationAnimaliaColeopteraStaphylinidae

B095B9DD-DBB4-5AD5-8693-B73FC6D8B2E1

https://zoobank.org/61B0186C-9346-478C-B8A8-03A17F98194B

Figs 5C, D , 6E–H

##### Type material.

***Holotype*** • ♂ (MECN-EN 23776): “Ecuador. Napo, Archidona. Pacto Sumaco, -0.658219, -77.59197. Malaise, 22-ene-2024. A Pazmiño | M Barreno” / “Caterino DNA voucher, Ext. MSC-13222, Morphosp. PS.A.004” / “MECN-EN 23776”. ***Paratypes*** (3♂ 1♀) • same data as type (MECN-EN 23774, 23777, 23775, 23778).

**Figure 6.**
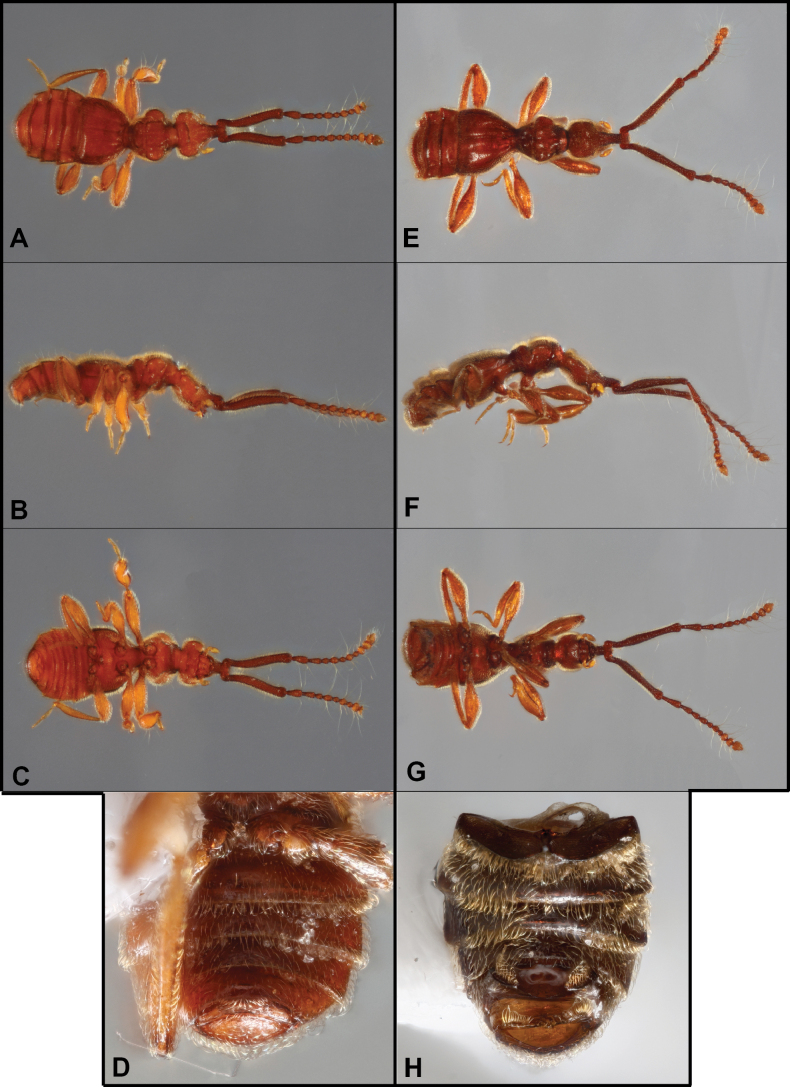
Habitus of *Bibrax* species (A, E. Dorsal view; B, F. Lateral view; C, G. Ventral view; D, H. Male abdominal ventrites). A–D. *B. canelazo* sp. nov.; E–H. *B. pectinifer* sp. nov.

##### Diagnosis.

BL = 1.55 mm (*n* = 3). Body densely setose, brownish-orange; eyes of both sexes strongly reduced, borne on small prominences, comprising three or four indistinct ommatidia; head rounded posteriorly, prolonged in front of eyes to prominent antennal base, two deep depressions in lateral view: one in front of eyes and another one at vertexal fovea; antennal scape slightly sinuate, narrowed anterior to apex, antennomere II (pedicel) longer than antennomeres III–IV combined, III–VI progressively shorter, VII slightly larger, VIII small, IX and X similar sized, IX–XI forming club, antennomere XI rounded and slightly longer than antennomeres IX and X; gular teeth not prominent; pronotum with lateral lobes slightly produced, rounded, pronotum weakly constricted and gradually narrowed to base; median and lateral longitudinal pronotal impressions well developed; elytra rather short, with narrowed humeri, each with sutural and one lateral basal foveae, lateral fovea with broad longitudinal impression extending posterad; abdominal paratergites wide, projecting at posterior corners; penultimate male abdominal ventrite weakly depressed at middle with comb-like series of erect setae along apicolateral margins; last ventrite with similar setal comb less prominent. Aedeagus (Fig. 5C, D) with basal bulb large and spherical, with pair of short, blunt basal apodemes; tegmen deeply divided into two separate processes of similar lengths, one sinuate, with concave apical margin, the other with spine-like end and curved dorsally.

##### Distribution.

This species is known only from the lower (~1500 m), southern slopes of the Volcán Sumaco, in the western Amazon, Napo Province, Ecuador.

##### Remarks.

This species is among the smaller, flattened species, and accordingly differs mainly in the male abdominal characters. The paired combs of setae on the last two male ventrites are unique and readily distinguish the species, although these combs may be somewhat obscured by adhering particles of dirt.

##### Etymology.

We name this species for its males’ ‘comb-bearing’ abdominal sternites.

#### ﻿ Bibrax arachnoides sp. nov.

Taxon classificationAnimaliaColeopteraStaphylinidae

1C80667B-A7EB-59C3-B427-704DD50338B4

https://zoobank.org/6AFD0837-D4F1-4DA9-8B91-635EF30FC184

Figs 5E, F , 7A–D

##### Type material.

***Holotype*** • ♂ (MECN-EN 40778): “Ecuador: Pastaza, -1.4267, -78.0439, Rio Anzu Reserve, 1342m, 11.XI.2024, M.Caterino, sifted litter” / “Caterino DNA voucher, Ext. MSC-13131, Morphosp. RAn.014” / “MECN-EN 40778”. ***Paratypes*** (6♂, 1♀) • Ecuador: Pastaza, Sumak Kawsay in situ Reserve, 26-JAN-2024, Díaz-Guevara (MECN-EN 23781-23787)

##### Diagnosis.

BL = 1.68 mm (4). Body densely setose, densely and finely punctate beneath; head rounded at base, vertexal foveae shallowly impressed, short vertexal tubercles present at base; frons narrowing and rising to common antennal base, with shallow median sulcus on surface; eyes (of male) strongly reduced, comprising short longitudinal series of two or three indistinct facets; gular teeth well-developed; antennae with scape very long, slightly sinuate, antennomere II (pedicel) slightly longer than II–V combined, in male slightly convex on outer margin, produced at inner apical corner, III–VIII all bead-like, but V and VII larger and bearing elongate setae, IX–XI forming loose club, X and XI slightly more closely associated than IX and X; pronotum elongate, ~1.25× longer than wide, widest near front, sides rounded; pronotal disk with lateral and median subbasal foveae; disk with only weak lateral longitudinal impressions; elytra rather short (wingless), humeri evenly sloped, sides rounded, each elytron with sutural and lateral basal foveae, lateral fovea with strong impression running posterad ~1/3 elytron length; subhumeral fovea absent; protibia swollen; male metatrochanter with inner margin expanded, subquadrate, distal corner projecting perpendicularly as small, truncate tooth; last male abdominal ventrite weakly depressed, bearing small basal marginal tooth and larger median apical marginal flange; penultimate male ventrite not obviously modified. Aedeagus (Fig. 5E, F) with subspherical basal bulb, bearing large, round dorsal diaphragm; basal apodemes paired, thin, slightly curved; tegmen short, widened from base to middle, sclerotized portion bent strongly ventrad into a hook with deeply divided, densely bristled lobes extending laterodistad; between them an unsclerotized, setose median lobe extends from tip.

**Figure 7.**
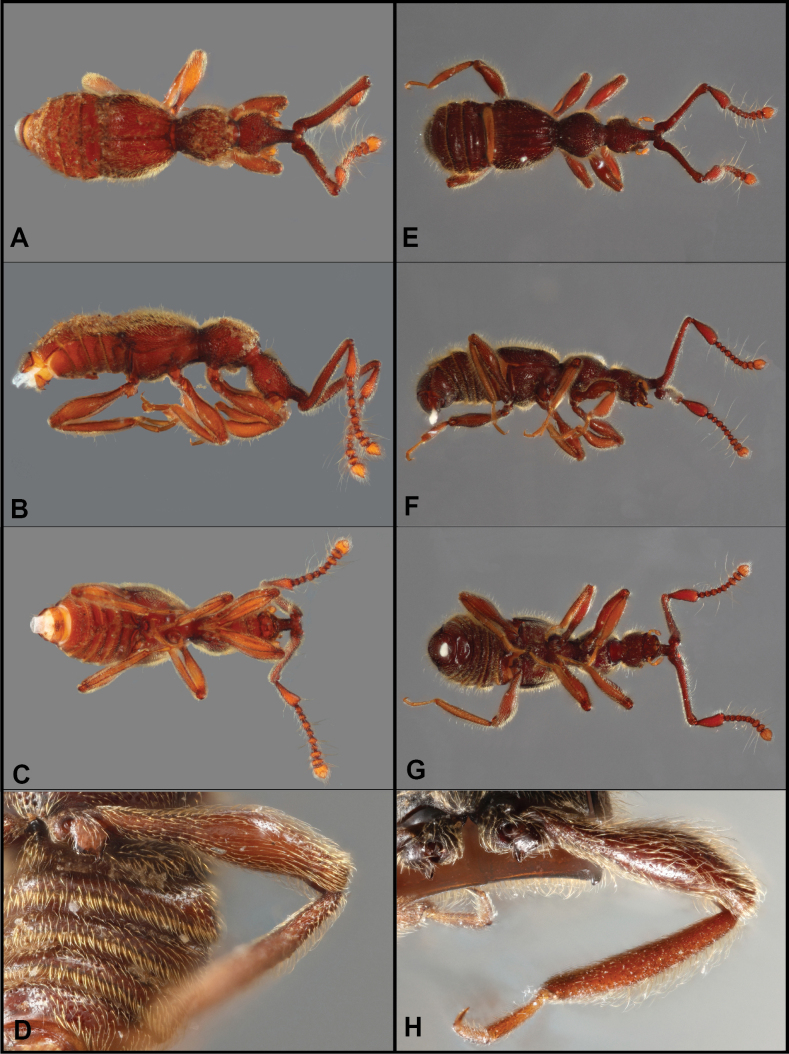
Habitus of *Bibrax* species (A, E. Dorsal view; B, F. Lateral view; C, G. Ventral view; D, H. Male metatrochanter). A–D. *B. arachnoides* sp. nov.; E–H. *B. amasanga* sp. nov.

##### Distribution.

This species is known from two localities in the foothills of the Andes, in western Pastaza Province, Ecuador.

##### Remarks.

This species and the next two are very similar and share the probable synapomorphy of a swollen antennomere II. They may share this synapomorphy with *B. chullachaqui*, which is otherwise quite distinct in its large eyes, short body setae, and various other characters. *Bibrax arachnoides* has slightly different modifications of the last male abdominal ventrite (with a small basal tooth), metatrochanter (more generally subquadrate along apical margin with a perpendicular distal tooth), and antennomere II (more or less symmetrically swollen). The male genitalia of all these species are unambiguously distinct.

##### Etymology.

We name this species for the somewhat spider-like appearance, common to members of *Bibrax*, as a result of their geniculate antennae appearing to be an extra pair of legs.

#### ﻿ Bibrax amasanga sp. nov.

Taxon classificationAnimaliaColeopteraStaphylinidae

00DE5EF1-53B1-5290-AEDC-4BD1012614FD

https://zoobank.org/7BE0CC78-69DE-4217-910D-40936331B4F7

**Figure 8.**
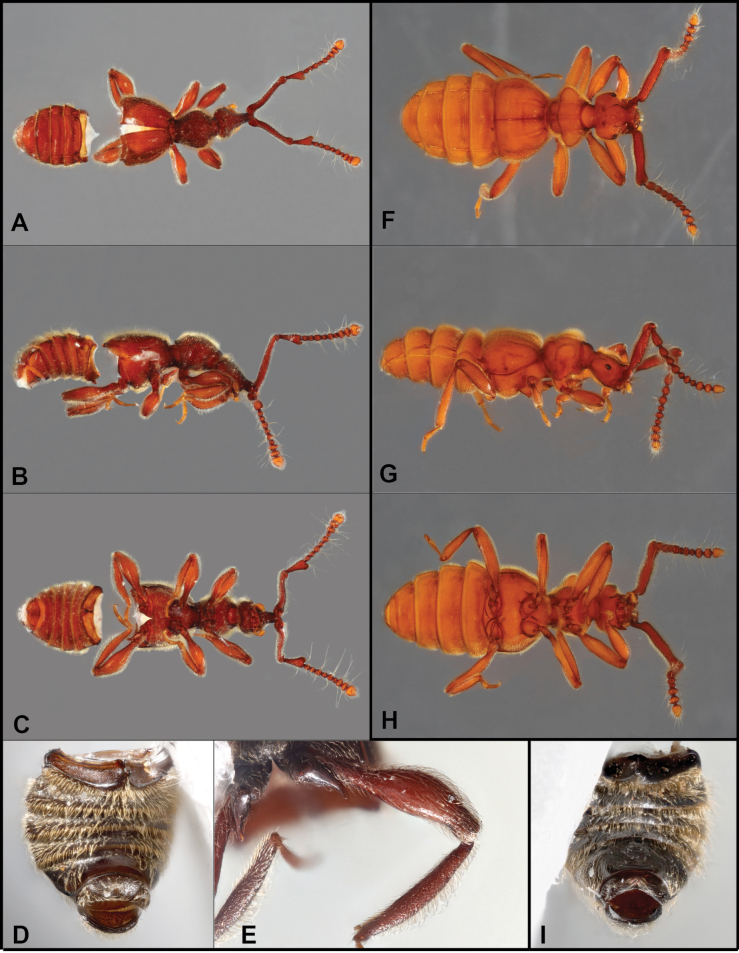
Habitus of *Bibrax* species (A, F. Dorsal view; B, G. Lateral view; C, H. Ventral view; D–I. Male metaventrite; E. Male metatrochanter). A–E. *B. yasuni* sp. nov.; F–H. *B. grandis* sp. nov.; I. *B. amasanga* sp. nov.

Figs 5G, H , 7E–H , 8I

##### Type material.

***Holotype*** • ♂ (QCAZ-I-278673): “Ecuador. Orellana, Estación Científica Yasuní PUCE, 496m, -0.68315, -76.40004, 12 - 2016. Winkler, Brian Four” / “Caterino DNA voucher, Ext. MSC-13224, Morphosp. 278673” / “QCAZ-278673”. ***Paratype*** • 1♂: “Ecuador F.co Orellana, Chiruisla Km0,2, 218m, 00°36'50"S, 75°52'34"W, 08-13DEC 2005, J. Vieira” / “Ex: Winkler trap, Primary forest” (QCAZ-I-280398).

##### Diagnosis.

BL = 2.09 mm (*n* = 1). Body densely setose and brown, finely punctate above; head rounded at base, vertexal foveae impressed, without vertexal horns or tubercles at base; frons narrowing and rising to common antennal base; eyes reduced, with two or three ommatidia; gular teeth well-developed; antennae with scape very long, slightly sinuate at base, antennomere II (pedicel) slightly longer than III–VI combined, globose, narrow at base, widening towards apex, convex along outer margin, slightly concave on inner, protruding slightly at apical internal corner, III–VIII all bead-like, V and VII slightly larger and bearing elongate setae, IX–XI forming loose club, IX and X bearing elongate setae, XI densely setose; pronotum slightly longer than wide, widest near front, sides rounded; pronotal disk with lateral and median subbasal foveae; disk with strongly marked lateral longitudinal impressions; elytra rather short (wingless), humeri evenly sloped, sides rounded, each elytron with sutural and lateral dorsobasal foveae, lateral fovea with strong impression running posterad ~2/3 elytron length; posterior margin of elytron with outer submarginal tooth; subhumeral fovea absent; dorsal surface of abdomen distinctly punctate; protibia swollen; junction of mesepimeron and metaventrite deeply foveate; metatrochanter with inner margin slightly expanded, with blunt tooth at inner corner; penultimate ventrite distinctly depressed at middle, swollen at sides, last abdominal ventrite bearing small median apical marginal flange. Aedeagus (Fig. 5G, H) with rather reduced basal bulb, slightly rounded dorsally, with oval dorsobasal diaphragm; basal apodemes forming wide elongate plate; tegmen not obviously articulated with basal bulb, dorsally subdivided into two asymmetric processes, one shorter, thinner strongly arched, the other longer, wider, curved ventrally to acute apex; additionally, below tegmen, two thin, curved, spine-like processes, project apically and basally from midpoint of tegmen, distal process flattened at base.

##### Distribution.

This species is known only from the Yasuní Biological Station in Amazonian Ecuador.

##### Remarks.

This species and the next are extremely similar in external morphology, and, coming from the same locality, were not initially considered to be distinct species. Both are also similar to the preceding species from the Rio Anzu area, ca 200 km west (and 1000 m higher in elevation). While the male genitalia separate these species clearly, distinguishing the two Yasuní species from each other based on external morphology is challenging (particularly given that both are known only from single males). In both, males have antennomere II asymmetrically swollen and the male metatrochanter apically dentate. In this species the penultimate male abdominal ventrite appears slightly lengthened and more distinctly depressed than in the following. The shape of the swollen second antennomere also differs slightly, being more distinctly swollen along the outer margin in this species, and less flanged at the inner apex. Also, the male metatrochanters are distinct, with that of *B. amasanga* basally toothed, that of *B. yasuni* markedly prolonged at the apex.

We have two female *Bibrax* specimens from Yasuní that appear to be distinct from each other. One of them was collected in the same sample as this species: the two individuals differ considerably in size, with a smaller one comparable in size to both *B. amasanga* and *B. yasuni* (described below). The second female specimen is significantly larger and differs in depth and length of the elytral impressions, as well as in minor details of antennomere lengths. At present it is impossible to associate either of these with either of the described species from this site: it is possible that the larger specimen represents yet a third species from the site. More material and possibly DNA sequences will be necessary to make definitive associations.

##### Etymology.

This species’ name refers to a mythical ‘spirit of the jungle’, Amasanga, who came to the people in dreams and taught them to hunt

#### ﻿ Bibrax yasuni sp. nov.

Taxon classificationAnimaliaColeopteraStaphylinidae

6C0A52DE-851F-54FC-A7E1-171010F196E4

https://zoobank.org/83FCB599-8446-41F4-A722-B64417EC1CA0

Figs 5I, J , 8A–E

##### Type material.

***Holotype*** • ♂ (QCAZ-I-278674): “Ecuador. Orellana, Estación Científica Yasuní PUCE, 253m, -0.68315, -76.40004, 09 - 2017. Winkler., A. Argoti & R. E. Cárdenas” / “Caterino DNA voucher, Ext. MSC-13225, Morphosp. 278674” / “QCAZ-278674”. ***Paratype*** • 1♂: “Ecuador: Yasuni National Park, Yasuni Biological Station, 0°40'32"S, 76°23'50"W” / “29 June 1999, Berlese, 15km West of station, Monkey Plot, CEC#034, CECarlton” (QCAZ-I-280395).

##### Diagnosis.

BL = 1.76 mm (*n* = 1). Body setose and reddish, finely punctate above; head rounded posteriorly, vertexal foveae slightly impressed; frons narrowing and rising to common antennal base; eyes reduced, with two or three ommatidia; gular teeth well-developed; scape elongate, weakly sinuate, antennomere II (pedicel) slightly longer than III–VI combined, narrow at base and widening towards distal end, with outer margin straight, inner apical angle protruding forming flattened, slightly translucent, rounded spatulate process, III-VIII bead-like, V and VII slightly longer and bearing elongate setae, antennomeres IX–XI forming loose club, IX and X slightly transverse and bearing elongate setae, XI densely setose; pronotum elongate, widest near front, sides rounded; pronotal disk with lateral and median subbasal foveae; disk with strongly marked lateral longitudinal impressions; elytra rather short (wingless), sides rounded, each elytron with sutural and lateral basal foveae, lateral fovea with strong impression running posterad; subhumeral fovea absent; outer posterior corner of each elytron with tooth; dorsal surface of abdomen largely impunctate; protibia swollen; metatrochanter with distal corner extended as strong spine; metafemora swollen, distinctly clavate; last abdominal ventrite bearing small median apical marginal flange; penultimate ventrite shorter, barely depressed at middle. Aedeagus (Fig. 5I, J) with round basal diaphragm, short and irregularly subspherical basal bulb; basal apodemes conjoined, broad, flat, with prolonged apical corners; tegmen narrow and tubular at base, expanded distally, dorsally hooded, with strong, blunt ventral keel, two distal processes that are similar in length extend from ventral apex, one thinner, slightly sinuate and apically subacute, the other wider, with basal brush of setae along outer margin, apically bifid with sclerotized, acute tip and secondary, setose, unsclerotized apical lobe; expansive membranous internal sac surrounding much of tegmen apex.

##### Distribution.

This species is known only from the Yasuní Biological Station in Amazonian Ecuador.

##### Remarks.

As discussed above, this species is very similar to the preceding. Aside from the distinctive male genitalia, *B. yasuni* is most distinct in the shape of antennomere II of the male, having a straight outer margin and flanged inner apical corner, the male penultimate abdominal ventrite, being shorter and barely depressed at the middle, and in the apically prolonged male metatrochanter.

##### Etymology.

This species is named for the Yasuní Biological Station, its type locality.

#### ﻿ Bibrax grandis sp. nov.

Taxon classificationAnimaliaColeopteraStaphylinidae

C4ACA30D-22C0-5DC4-B1D6-FC5EE544C4D4

https://zoobank.org/0F472765-D16D-4024-8DD3-A4E7A611316D

Fig. 8F–H

##### Type material.

***Holotype*** • ♀ (MECN-EN 40146): “Ecuador: Cotopaxi, -0.4168, -79.0044, Bosque Integral Otonga, 14.VIII.2024, 2093 m, M. Caterino | A. Pazmiño, Sifted leaf litter” / “Caterino DNA voucher, Ext. MSC-12889, Morphosp. Ot.A.054” / “MECN-EN 40146”.

**Figure 9.**
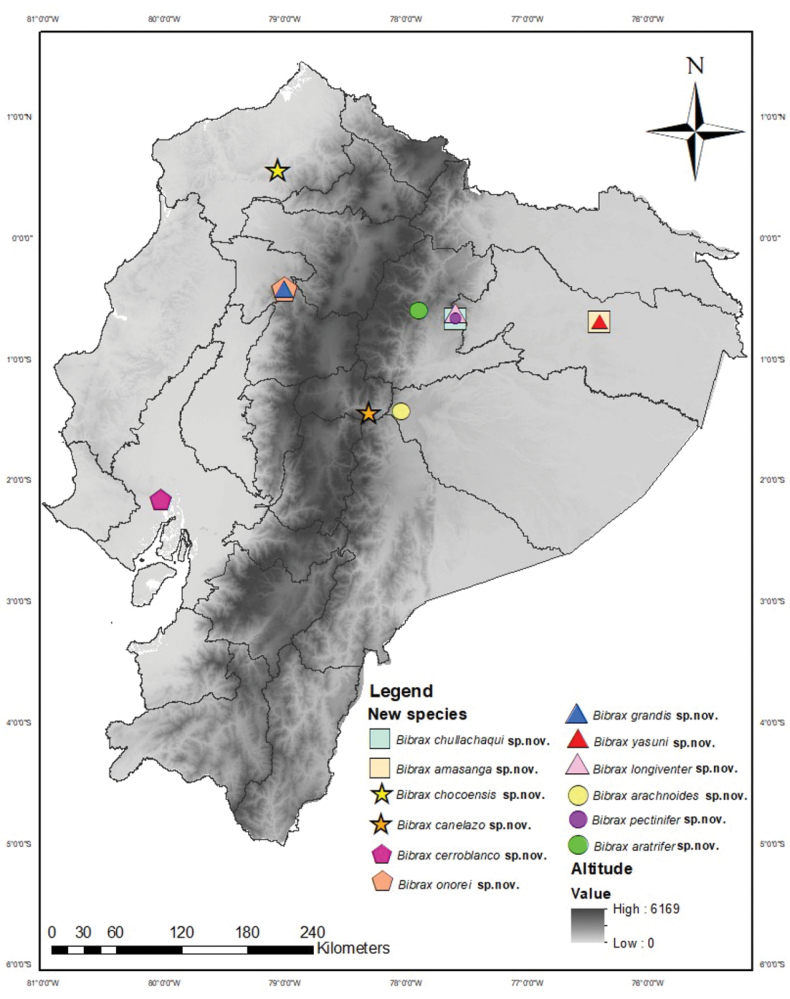
Map of all species localities of Bibrax in Ecuador.

##### Diagnosis.

BL = 1.73 mm (*n* = 1). Body large, very densely setose; eyes (of female) with multiple facets, head rounded at sides, short anterior to eyes, with pair of lyriform carinae; gular teeth well-developed; antennal tubercle short, wider apically; antennal scape cylindrical, coarsely punctate, antennomeres II–V more slightly enlarged and more densely setose than VI–X; pronotum with anterior lobes weakly angulate laterally, median and lateral longitudinal sulci deeply impressed; elytra moderately long, not together emarginate along posterior margin; dorsal elytral sulci (from mid-basal foveae) distinct, reaching just beyond elytral midpoint; abdominal tergites densely setose; posterior corners of abdominal laterotergites rounded.

**Male.** Unknown.

##### Distribution.

This species is known only from the higher elevations of the Bosque Integral Otonga, in northern Cotapaxi province, Ecuador.

##### Remarks.

This species is highly distinctive among known *Bibrax* species. Although known only from a single female, it is unusual enough to warrant recognition. It is most distinctive in its much shorter antennal base, lyriform frontal carinae, and produced subangulate pronotal sides. While the eyes are reduced, they exhibit a few more ommatidia than most of the reduced-eye species described here. The male might be expected to differ in eye size, and its discovery would also permit assessment of some other phylogenetically useful characters, such as presence of secondary sexual characters of the abdominal ventrites, mesotibiae, and metatrochanters.

##### Etymology.

While appearing related to many of the smaller, flattened species described in this study, this species is considerably larger than most of them, leading to our naming it *B. grandis*.

## ﻿Discussion

The species of *Bibrax* are remarkable among Metopiasini in their range of sexual dimorphisms. The most conspicuous are those species in which the males have fully developed eyes, while those of the females are reduced, such as *B. popeye* and *B. chocoensis*. This may also be the case for *B. chullachaqui*, for which we have only a large-eyed male specimen, and perhaps *B. grandis*, where the only female has more ommatidia than most of the other small-eyed species in the genus. This probable ancestral dimorphism has led to a reduction in the eyes of both sexes in the majority of the species.

Eye size and dimorphism seem to correspond in all cases to the presence/absence of wings, with the small-eyed forms wingless, regardless of sex. It is not certain, however, if this is the case in *B. bradleyi*, where the elytra of the male type appear (from photographs) rather large, with slightly wider humeri, suggesting flight ability. But the presence of wings cannot be determined and was not mentioned in the original description. Outside of *Bibrax* (and outside a few troglobitic species) most Metopiasini are fully winged, as far as is known, and this suggests a distinct ecological role for many of these species. While habits in the broader tribe are poorly documented, it has been suspected that many are myrmecophilous (Parker 2016; Chaul and Lopez-Andrade 2024). Whether such habits pertain to the deeper branching members of *Bibrax* remains to be seen.

Beyond sexual dimorphisms related to mobility, male secondary sexual characters may be found on several different parts of the body in *Bibrax*. In the original description of *B. bradleyi*, Fletcher (1927: 151) noted dimorphism in the last three male abdominal ventrites, being flattened or depressed, and similar modifications are common to many of the species we now recognize. Probably related to male maintenance of position on the female during mating, variation in these characters helps to recognize many of the species now described.

The male genitalia of the now-known species of *Bibrax* are remarkably varied and differ sharply from those described for other genera of Metopiasini. The open basal bulb with articulated dorsal tegmen is unusual, with something vaguely similar seen only in a few species of the otherwise dissimilar *Metopioxys* (such as *Metopioxys chandleri*; see Comellini 1983). The basal bulb within *Bibrax* shows particular variation in the basal apodeme(s), the portion articulating with the inner surface of the 6^th^ ventrite. The basal apodeme of *B. popeye* is shown to be rather large and internally open, while it is only a very rudimentary, bent tubercle in *B. bradleyi*. In several of the new species it is relatively simple (e.g., *B. chocoensis*), comparable to that of the latter species while in others it is in fact divided into a pair of basal apodemes (e.g., *B. canelazo*, *B. chullachaqui*). The basal bulb itself also varies considerably in size, while in *B. onorei* it is reduced to a distally open cup. A well-developed dorsal diaphragm is particularly conspicuous in *B. onorei*, but it may be more universally present in the genus, visible but not described in published figures of *B. popeye* and *B. bradleyi* (Asenjo et al. 2024), and definitely present in *B. aratrifer* and *B. longiventer*, though varied in how close to the distal tegmen it is.

The tegmen of these species also differs significantly, much thicker, straight, and with a brushy, truncate apex in *B. popeye*, whereas it is narrow, curved, and with a simply rounded apex in *B. bradleyi*. In several of the newly described species the tegmen is divided, shallowly in *B. chocoensis* and *B. cerroblanco*, and apparently completely in *B. longiventer*, where the two halves are highly differentiated. That of *B. onorei* is, again, difficult to homologize with any of these.

This study extends the known range of *Bibrax* southward, with species now known from Panama, Colombia, and Ecuador, a still rather limited distribution. While both previously known species were reported from lowland habitats (*B. bradleyi* from Gatun Lake Panama near sea level; *B. popeye* from far western Amazonia ca 300 m in Colombia), several of the new species described here extend the range upward, as high as 2500 m in the Andes. That most of these higher elevation species are flightless suggests a much richer fauna, with many species yet to be discovered in this highly dissected landscape. The Amazonian lowland fauna now also comprises several distinctive species, indicating much still to be found there. In general, the species seem to be most often collected by litter sifting, and not at lights or in flight traps, which may explain their apparent rarity. We may confidently predict that additional litter sampling will produce many additional species and will extend the range of the genus much further southward in the South American continent. We strongly encourage other workers to test this prediction.

## Supplementary Material

XML Treatment for Bibrax

XML Treatment for Bibrax chullachaqui

XML Treatment for Bibrax chocoensis

XML Treatment for Bibrax onorei

XML Treatment for Bibrax aratrifer

XML Treatment for Bibrax longiventer

XML Treatment for Bibrax cerroblanco

XML Treatment for Bibrax canelazo

XML Treatment for Bibrax pectinifer

XML Treatment for Bibrax arachnoides

XML Treatment for Bibrax amasanga

XML Treatment for Bibrax yasuni

XML Treatment for Bibrax grandis

## ﻿Additional information

### Use of AI

No use of AI was reported.

### Author ORCIDs

Yarina Tapuy-Avilés https://orcid.org/0009-0001-2357-7916

David R. Díaz-Guevara https://orcid.org/0009-0001-4632-4460

Michael S. Caterino https://orcid.org/0000-0002-2597-5707

### Data availability

All of the data that support the findings of this study are available in the main text.
